# Supplementary material for: Effects of Darbepoetin Alfa and Ferric Derisomaltose Plus Darbepoetin Alfa in Functional Iron-Deficiency Anemia
Source: Int J Mol Sci. 2025 Feb 28;26(5):2203. doi: 10.3390/ijms26052203 (PMC11899774; doi:10.3390/ijms26052203)
Supplement: Supplementary file 1 [file ijms-26-02203-s001.zip › ijms-3490939-supplementary/ijms-3490939-supplementary.pptx]

## Slide 1
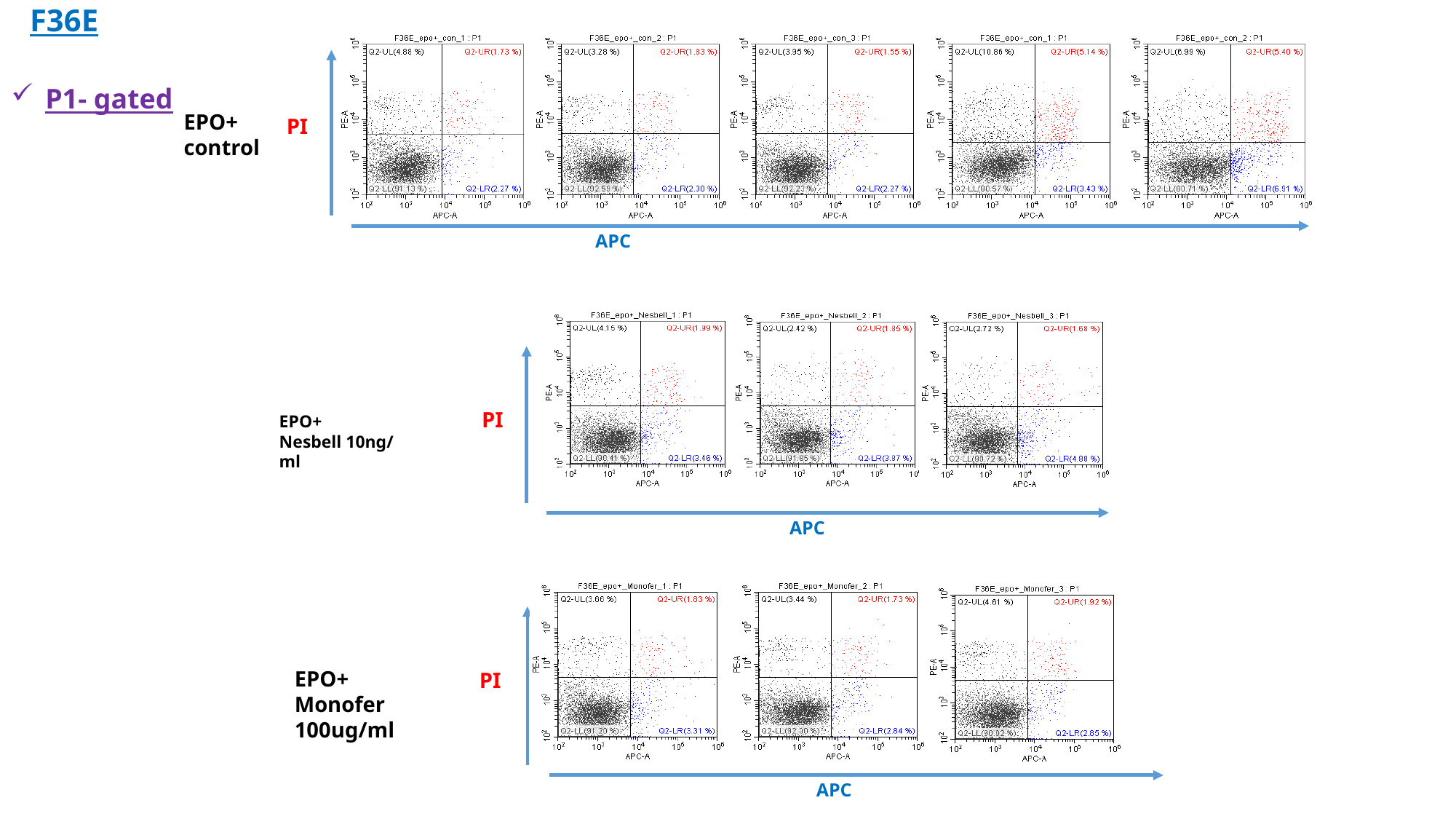

F36E
PI
APC
P1- gated
EPO+ control
PI
APC
EPO+
Nesbell 10ng/ml
PI
APC
EPO+
Monofer 100ug/ml

## Slide 2
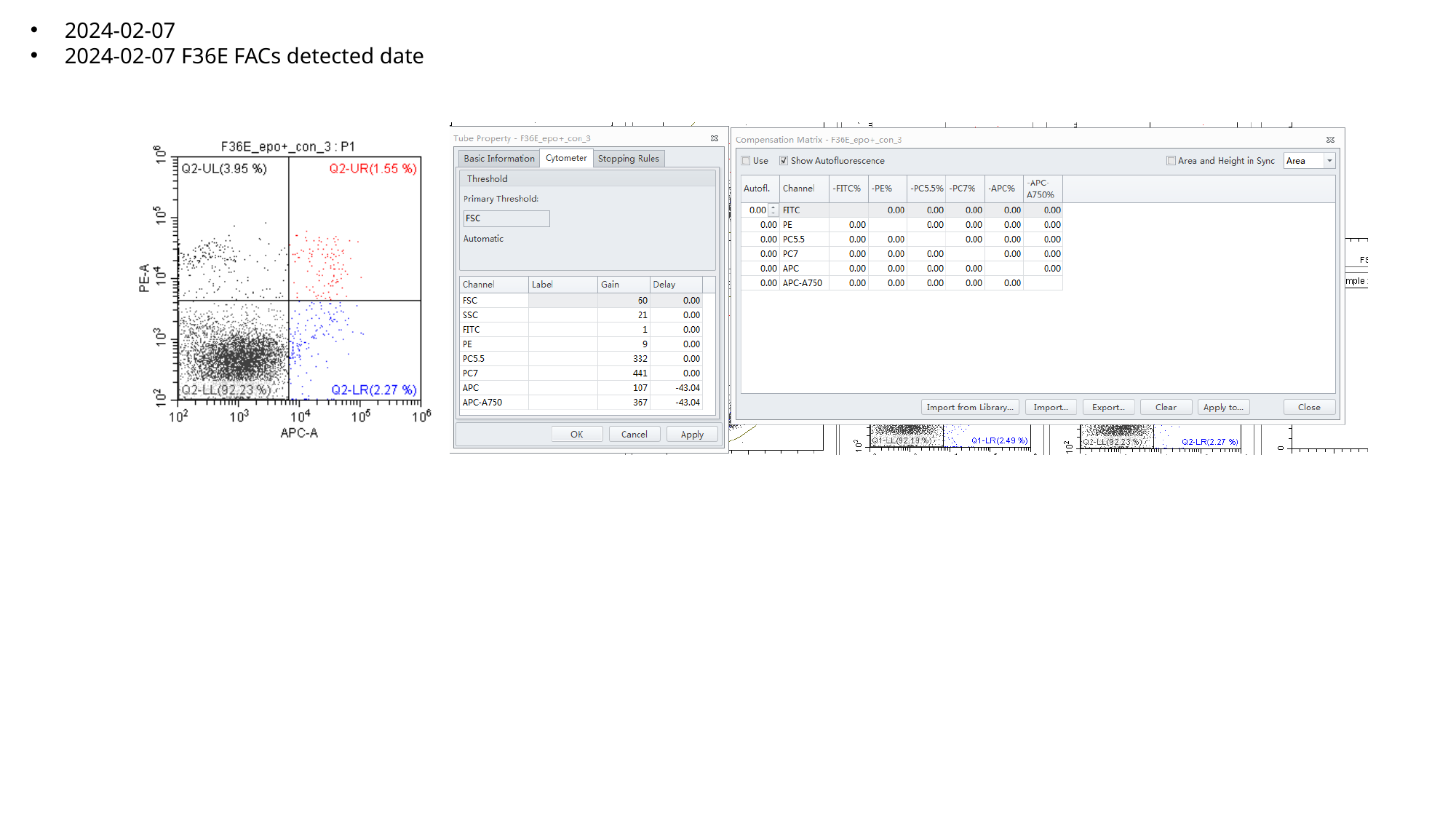

2024-02-07
2024-02-07 F36E FACs detected date

## Slide 3
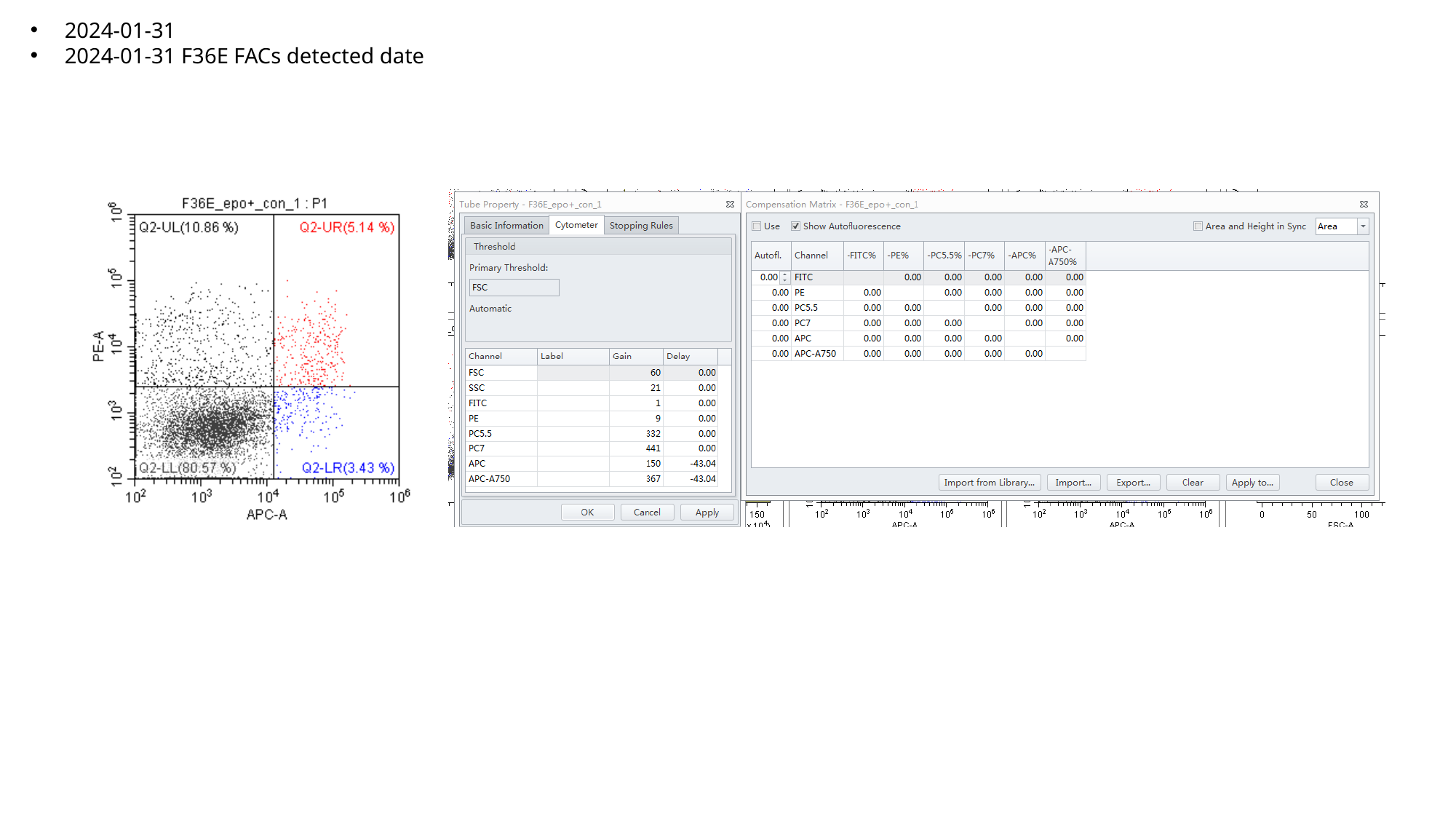

2024-01-31
2024-01-31 F36E FACs detected date

## Slide 4
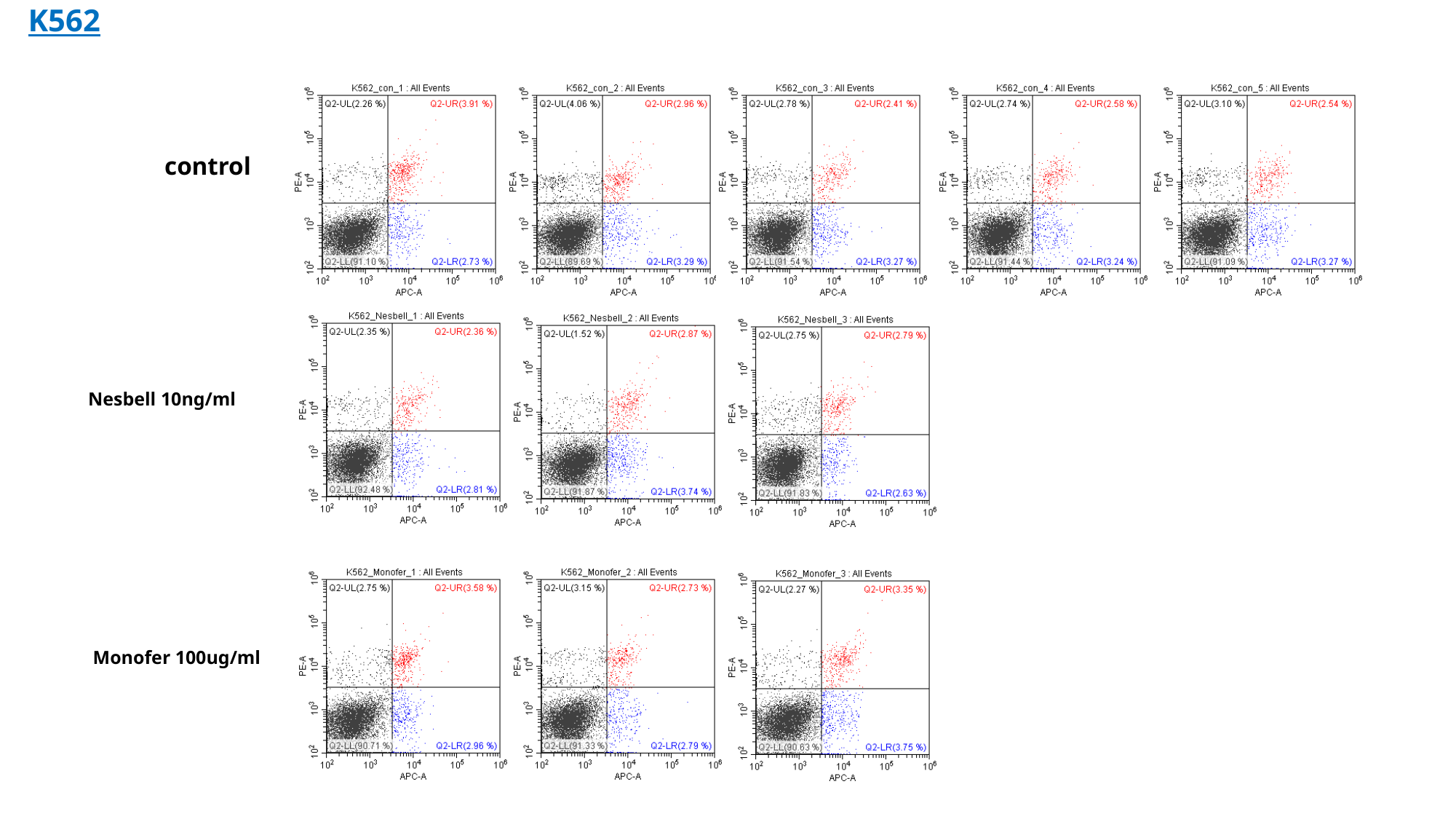

K562
control
Nesbell 10ng/ml
Monofer 100ug/ml

## Slide 5
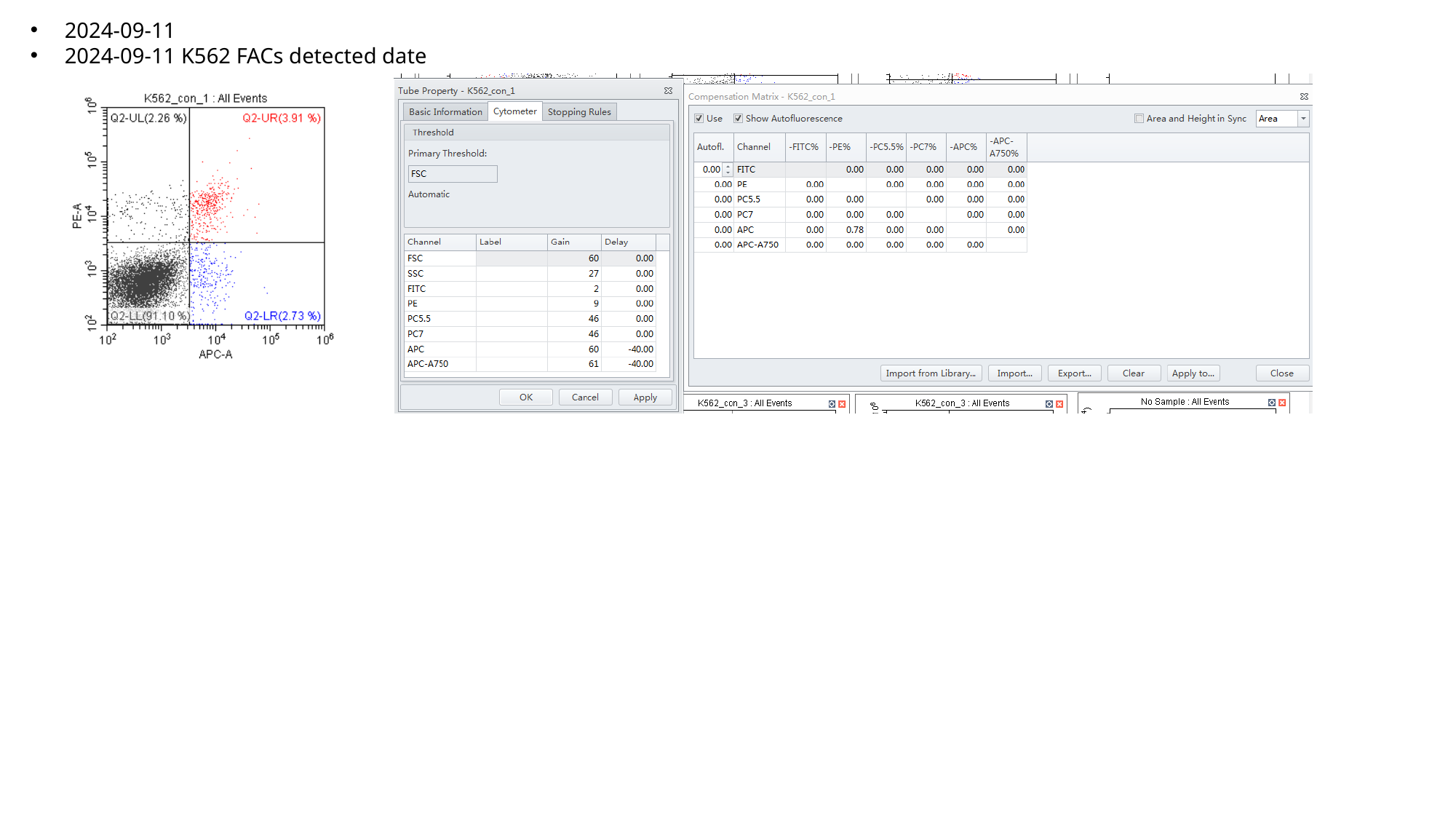

2024-09-11
2024-09-11 K562 FACs detected date

## Slide 6
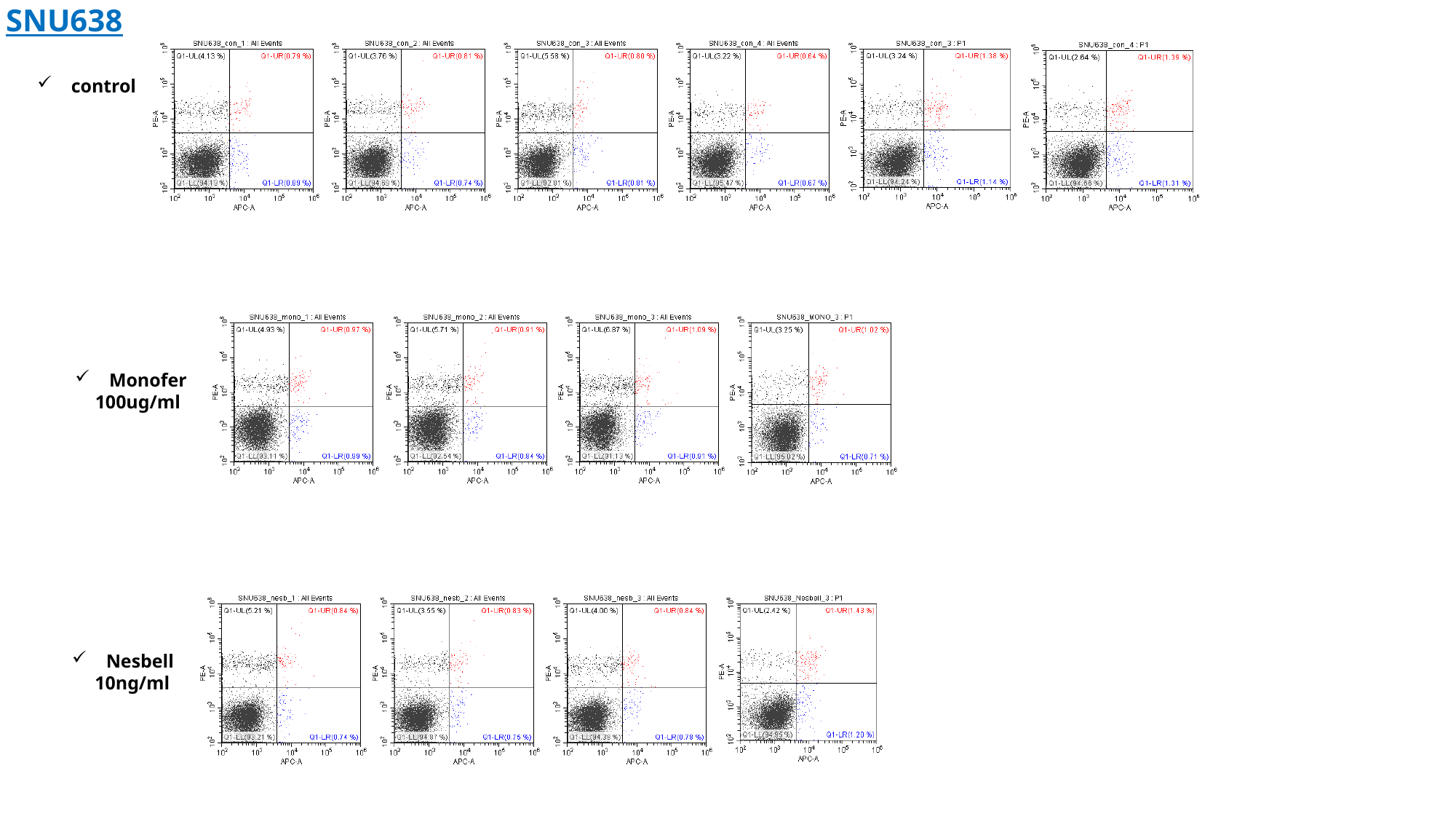

SNU638
control
Monofer
 100ug/ml
Nesbell
 10ng/ml

## Slide 7
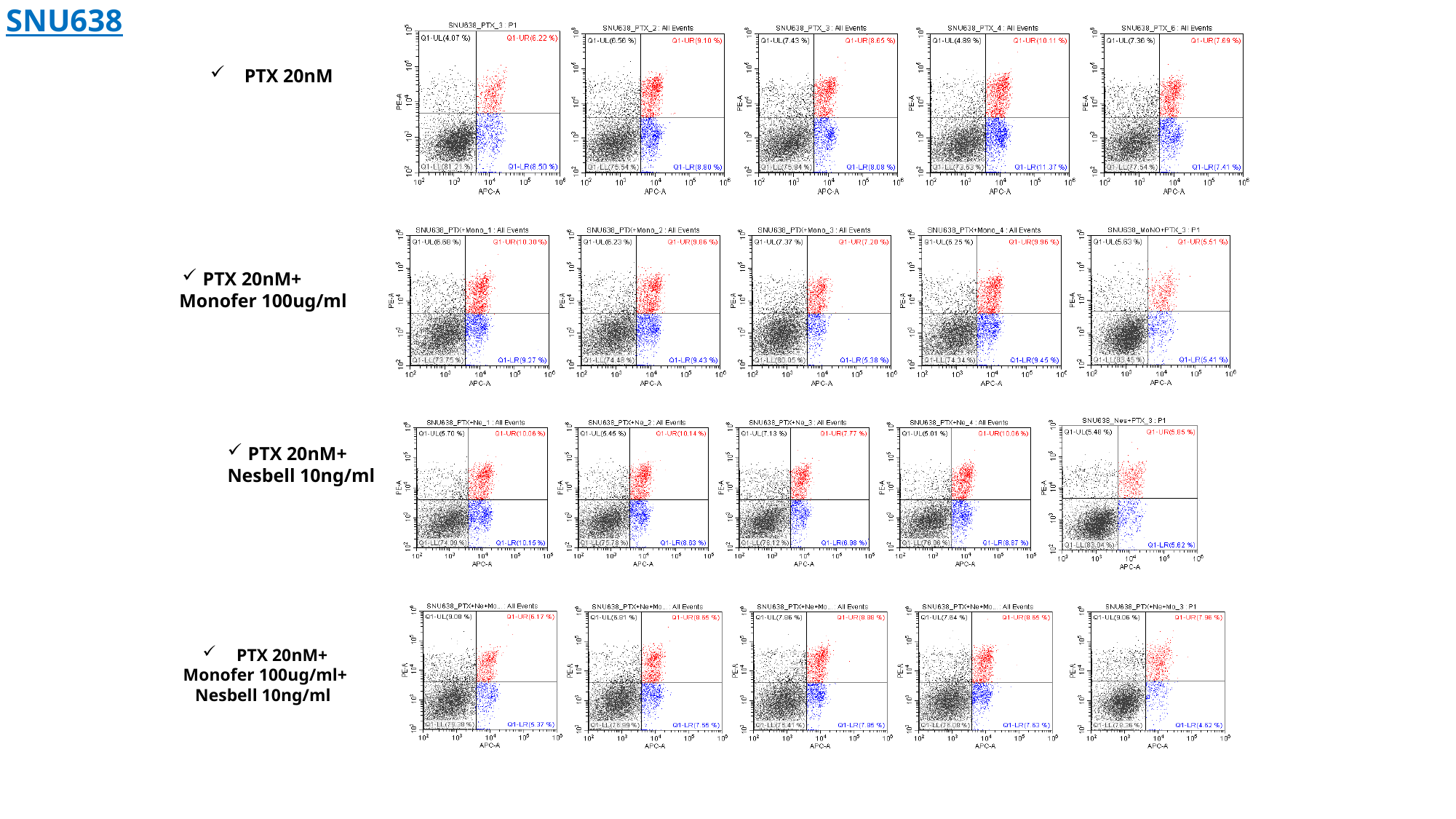

SNU638
PTX 20nM
PTX 20nM+
 Monofer 100ug/ml
PTX 20nM+
 Nesbell 10ng/ml
PTX 20nM+
Monofer 100ug/ml+
Nesbell 10ng/ml

## Slide 8
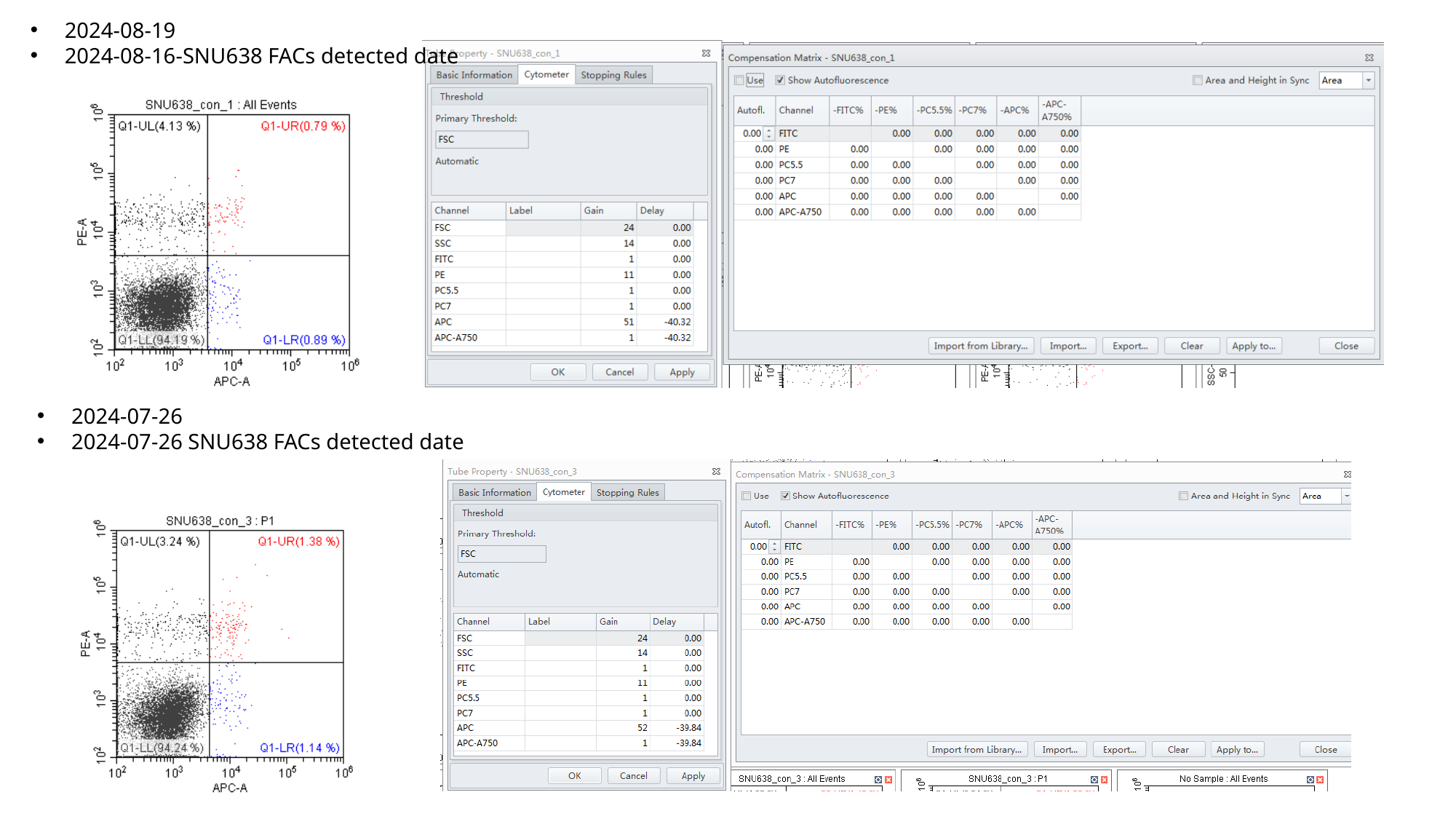

2024-08-19
2024-08-16-SNU638 FACs detected date
2024-07-26
2024-07-26 SNU638 FACs detected date

## Slide 9
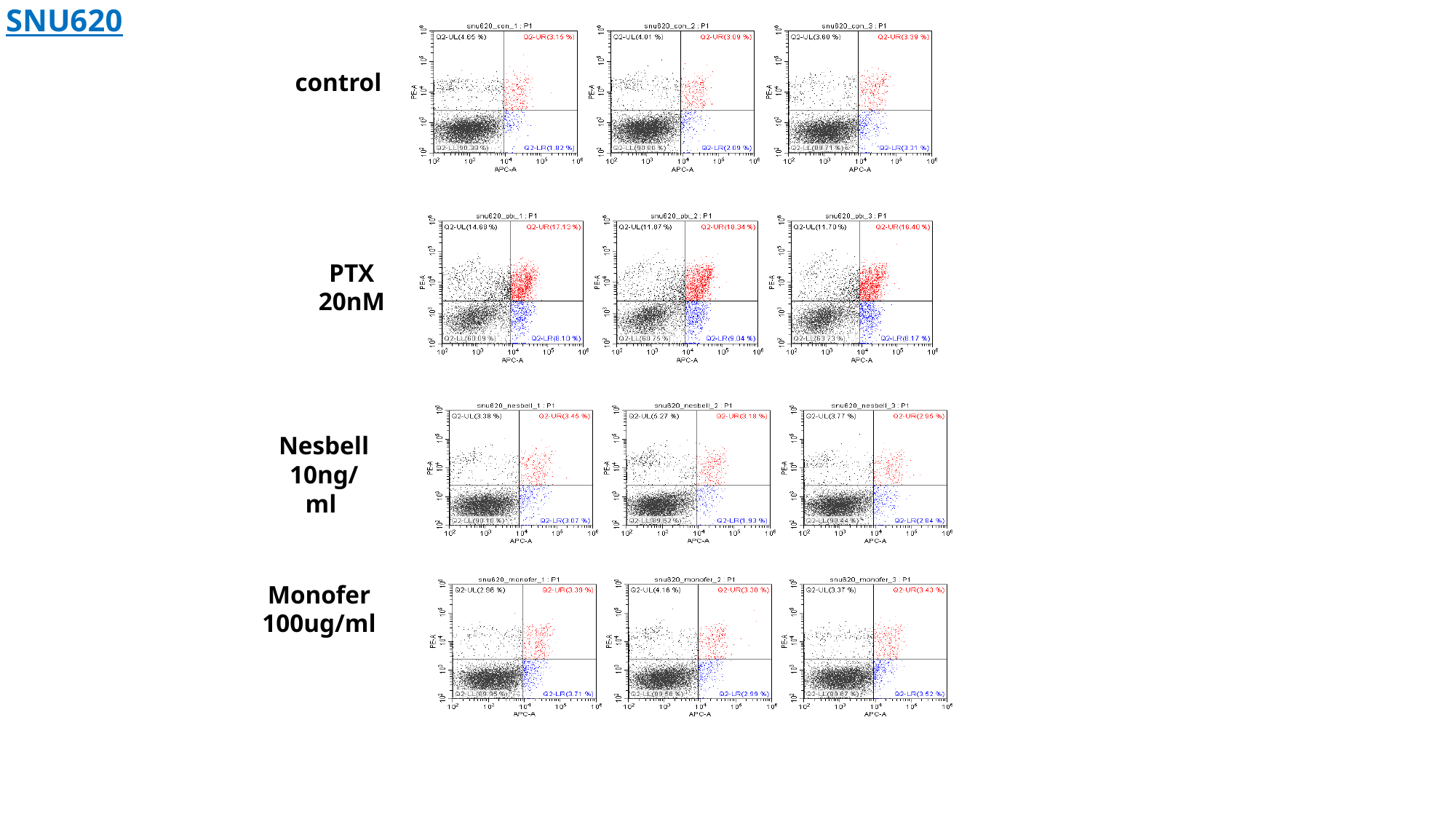

SNU620
control
PTX 20nM
Nesbell
10ng/ml
Monofer
100ug/ml

## Slide 10
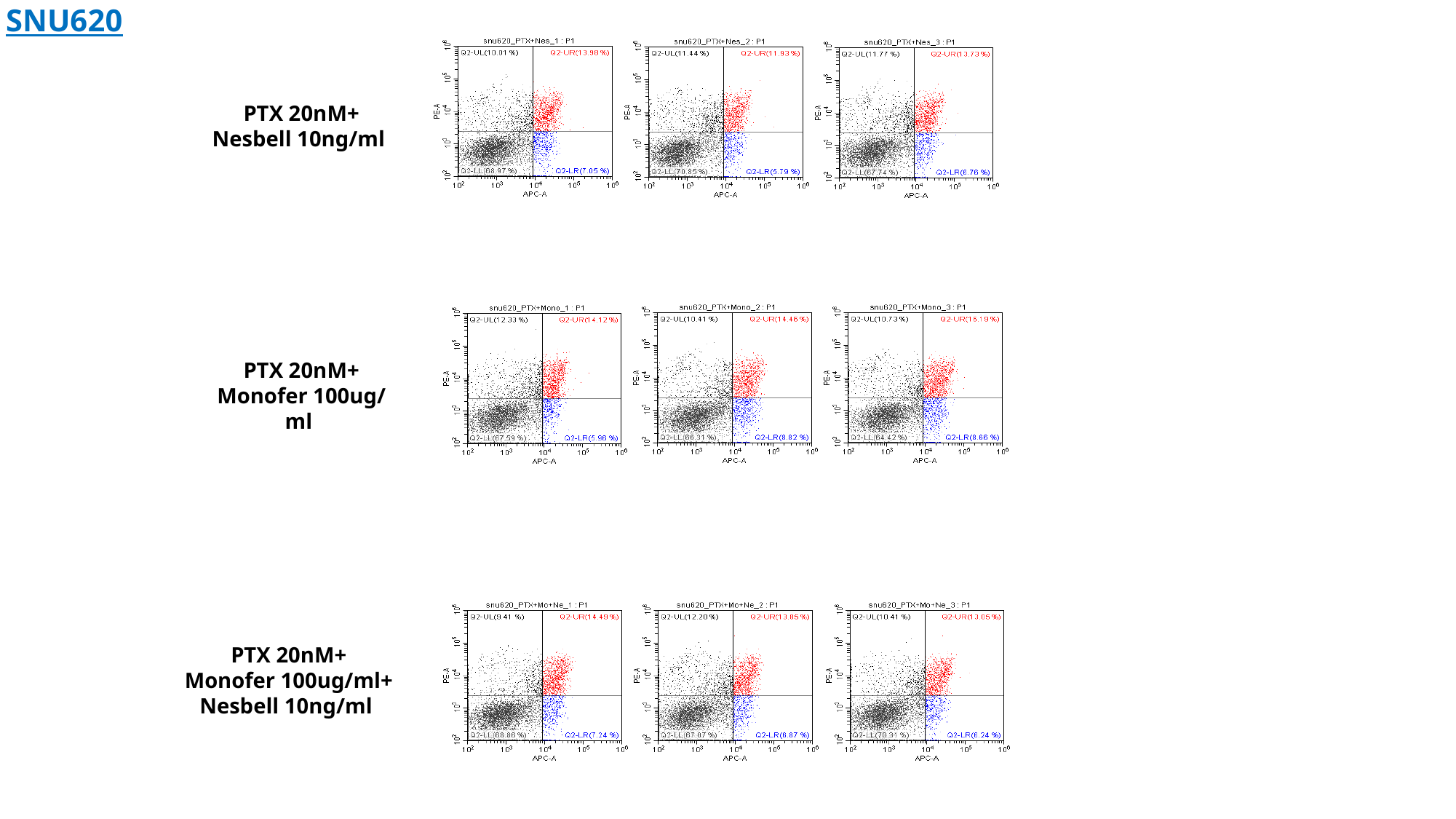

SNU620
PTX 20nM+
Nesbell 10ng/ml
PTX 20nM+
Monofer 100ug/ml
PTX 20nM+
Monofer 100ug/ml+
Nesbell 10ng/ml

## Slide 11
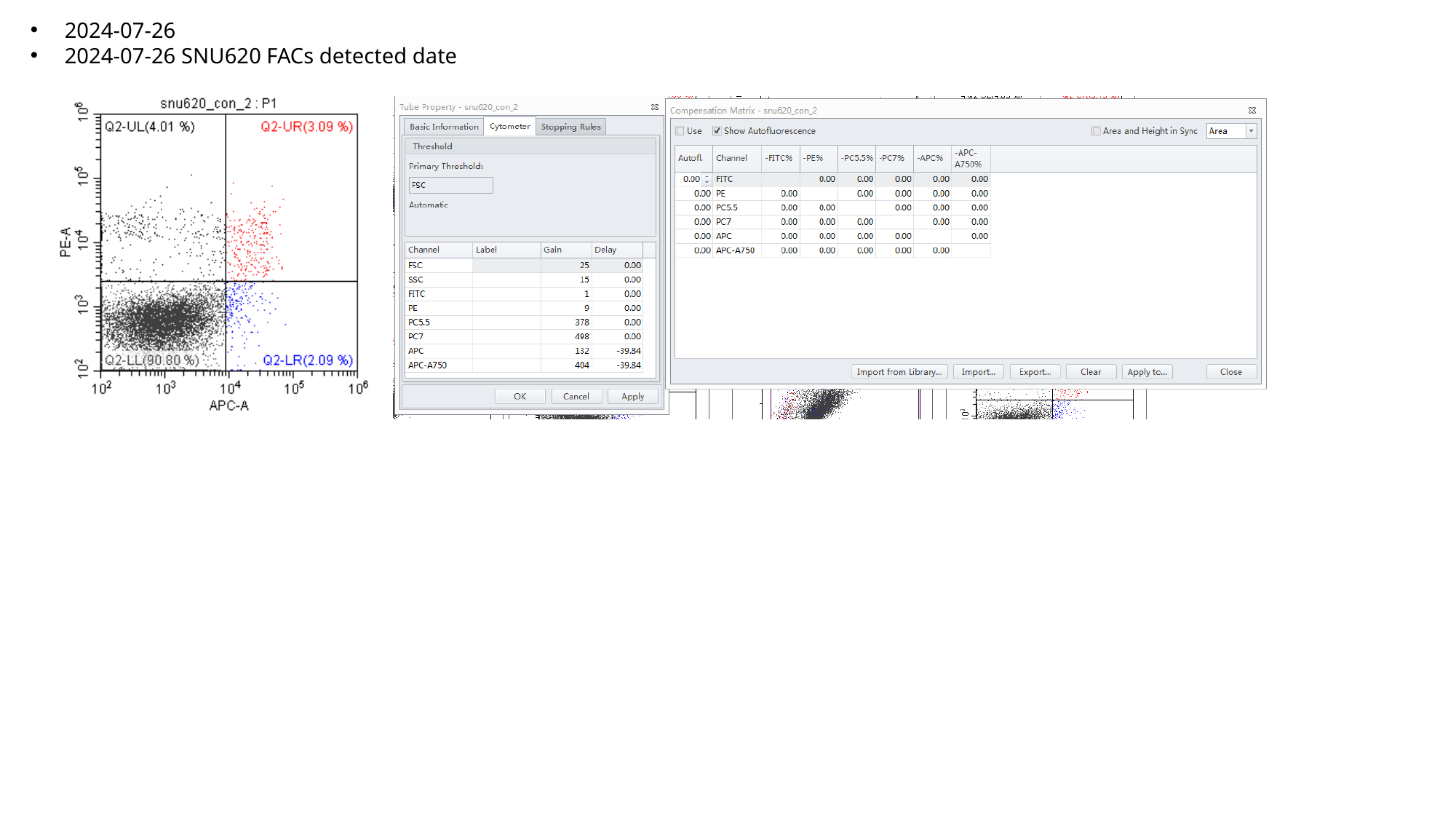

2024-07-26
2024-07-26 SNU620 FACs detected date
